# Development and evaluation of machine learning models for predicting relapse in idiopathic nephrotic syndrome

**DOI:** 10.3389/fendo.2026.1687315

**Published:** 2026-04-13

**Authors:** Mingrui Zhao, Ping Li, Fen Chen, Zhou Wu

**Affiliations:** Department of Kidney Disease, General Hospital of Central Theater Command, Wuhan, Hubei, China

**Keywords:** idiopathic nephrotic syndrome, machine learning models, neural networks, recurrence prediction, serological markers

## Abstract

**Background and aim:**

Idiopathic nephrotic syndrome (INS) is a glomerular disorder characterized by proteinuria, hypoalbuminemia, and edema, and relapse remains a major clinical challenge. Early prediction of relapse risk may facilitate individualized treatment and follow-up. This study aimed to develop and compare the performance of logistic regression, random forest, and deep learning models for predicting relapse in adult patients with INS using baseline clinical and laboratory data.

**Methods:**

We conducted a retrospective cohort study of 562 adult patients with idiopathic nephrotic syndrome treated between January 2022 and January 2024. The primary outcome was the first relapse within 12 months after baseline assessment. Baseline demographic characteristics, clinical history, laboratory parameters, and treatment-related variables were collected. The dataset was randomly divided into training (70%), validation (15%), and test (15%) sets. Missing data were imputed, continuous variables were standardized as appropriate, and SMOTE was applied to the training set only to address class imbalance. Three predictive models were developed: logistic regression, random forest, and a deep learning-based neural network. Model performance was evaluated using AUC, accuracy, sensitivity, specificity, and F1-score.

**Results:**

Among the three models, the deep learning model showed the best predictive performance, with AUCs of 0.908, 0.900, and 0.883 in the training, validation, and test sets, respectively. The logistic regression model showed intermediate performance, whereas random forest showed the lowest discriminatory ability. The most influential predictors of relapse included steroid resistance, nephrotic-range proteinuria at baseline, prior relapse history/frequency, elevated ESR, and immunosuppressant use.

**Conclusions:**

Deep learning demonstrated better predictive performance than logistic regression and random forest for predicting 12-month relapse in adult patients with idiopathic nephrotic syndrome. These findings suggest that machine learning-based models, particularly deep learning, may serve as useful tools for relapse risk stratification. External validation in larger independent cohorts is needed before clinical implementation.

## Introduction

Idiopathic nephrotic syndrome (INS) is a common glomerular disorder characterized by massive proteinuria, hypoalbuminemia, hyperlipidemia, and edema (1, 2). It remains an important cause of chronic kidney disease (CKD) and end-stage renal disease (ESRD) in both pediatric and adult populations. Despite advances in treatment, INS is prone to relapse, and many patients experience multiple relapse episodes during the course of disease. Recurrent relapse is clinically important because it may lead to repeated hospitalizations, prolonged exposure to corticosteroids and immunosuppressive agents, and an increased risk of complications such as thromboembolic events, infections, and treatment-related adverse effects (3, 4). In addition, frequent relapse may contribute to long-term renal injury and substantial healthcare burden. Therefore, the ability to accurately identify patients at higher risk of relapse is essential for optimizing follow-up strategies, supporting individualized treatment decisions, and improving long-term clinical outcomes.

Previous studies have explored a wide range of demographic, clinical, biochemical, and genetic factors associated with relapse risk in INS. Traditional statistical approaches, particularly logistic regression, have been widely used for risk factor identification and clinical prediction modeling. Several laboratory and clinical variables, including urinary protein levels, serum biochemical markers, lipid parameters, kidney function, treatment response, and prior relapse history, have been reported to be associated with disease activity and relapse risk (5, 6). However, conventional statistical models may be limited in their ability to capture complex, multidimensional, and potentially non-linear interactions among predictors, especially when multiple correlated clinical variables are considered simultaneously. In recent years, machine learning (ML) methods have attracted increasing attention in medical predictive analytics because of their ability to process high-dimensional data and detect complex patterns that may not be fully captured by traditional regression-based approaches (7–9). ML models such as random forests have shown promising performance in disease classification and outcome prediction tasks across a range of clinical settings (10). In nephrology, previous studies, including meta-analytic evidence, have suggested that ML-based prediction models may offer useful discriminatory performance for kidney disease progression and prognosis. Nevertheless, the application of deep learning approaches, particularly neural network-based models, to the prediction of INS relapse remains relatively limited, and comparative evidence in adult INS cohorts is still insufficient.

To address this gap, the present study aimed to develop and compare the predictive performance of logistic regression, random forest, and deep learning models for relapse prediction in adult patients with INS using baseline clinical and laboratory data. In addition to comparing overall model performance, we sought to identify the most influential predictors contributing to relapse risk and to evaluate whether more flexible ML-based approaches could improve prediction beyond conventional statistical modeling. By providing a systematic comparison of these approaches in a clinically relevant INS cohort, we hope to contribute evidence that may support future risk stratification strategies and inform subsequent validation and clinical translation efforts.

## Materials and methods

### Study design and data collection

This retrospective cohort study included 562 adult patients diagnosed with idiopathic nephrotic syndrome (INS) at the General Hospital of Central Theater Command between January 2022 and January 2024. The diagnosis of INS was established on the basis of standard clinical criteria and routine nephrology evaluation. Eligible patients were required to be at least 18 years old and to have complete baseline clinical data together with follow-up information available for at least 12 months after baseline assessment. Patients with secondary nephrotic syndrome, incomplete medical records, or loss to follow-up during the predefined observation period were excluded.

The study was conducted in accordance with the Declaration of Helsinki and was approved by the Institutional Review Board of the General Hospital of Central Theater Command. Because of the retrospective design, all data were collected from existing medical records and analyzed in a de-identified manner.

### Outcome definition and follow-up

The primary outcome was the occurrence of the first clinical relapse within 12 months after baseline assessment. Relapse was defined according to adult clinical criteria as the recurrence of nephrotic-range proteinuria (>3.5 g/day) following a period of remission. Relapse confirmation was based on clinical evaluation, laboratory findings, and urinalysis. Only the first relapse event during the 12-month follow-up period was considered for model development and evaluation. Patients without relapse during this period were classified as non-relapse cases.

At baseline, patients were categorized according to disease status as either experiencing their first episode of nephrotic syndrome or having a history of relapse. The number of relapses prior to baseline was recorded for each patient. Patients were also classified as frequently relapsing nephrotic syndrome (FRNS) or steroid-dependent nephrotic syndrome (SDNS) according to established adult clinical criteria when applicable.

All patients underwent a structured follow-up protocol during routine clinical care. In the first six months after baseline, follow-up assessments were generally performed monthly and included clinical evaluation and urinalysis. Between six and twelve months, follow-up visits were typically conducted every three months, with laboratory testing performed as clinically indicated. Although some patients continued longer-term clinical follow-up beyond 12 months, only events occurring within the predefined 12-month follow-up window were used for the present predictive modeling analysis.

### Candidate predictors and variable definitions

All candidate predictors were defined using information available at baseline. The dataset included demographic characteristics, clinical history, laboratory parameters, and treatment-related variables collected at the time of baseline clinical and laboratory assessment. Candidate predictors included age, body mass index, disease status at baseline, prior relapse history and frequency, steroid resistance, baseline proteinuria, kidney function, inflammatory markers, and baseline immunosuppressive therapy.

Several continuous variables were categorized using clinically established thresholds to improve interpretability and facilitate clinical application. Elevated erythrocyte sedimentation rate (ESR) was defined as >20 mm/hour, and elevated C-reactive protein (CRP) was defined as >5 mg/dL. Kidney function and proteinuria were categorized using standard nephrology practice where appropriate. The potential loss of information resulting from categorization was acknowledged as a limitation.

Baseline immunosuppressive therapy was defined as any immunosuppressive treatment being administered at the time of baseline clinical and laboratory assessment. Information on treatment type, including corticosteroids, calcineurin inhibitors, mycophenolate mofetil, cyclophosphamide, and combination therapy, was systematically recorded. These baseline treatment variables were considered candidate predictors. In contrast, immunosuppressive therapy initiated or modified after baseline was not included in the primary models, because post-baseline treatment decisions may be influenced by early clinical course and could introduce reverse causation or treatment–outcome coupling.

Nephrotic-range proteinuria (>3.5 g/day) was included as a baseline predictor to reflect disease severity. Although serum albumin was collected as part of routine clinical assessment, it was not included in the primary predictive models because baseline albumin levels may be influenced by albumin infusion, particularly in patients with severe hypoalbuminemia, which could introduce measurement bias. Proteinuria was therefore selected as a more stable indicator of nephrotic activity at baseline.

Genetic screening for monogenic forms of nephrotic syndrome was performed as part of routine clinical evaluation when clinically indicated. Patients with identified pathogenic or likely pathogenic variants associated with genetic nephrotic syndrome were excluded to ensure a more homogeneous idiopathic nephrotic syndrome cohort.

The unit of analysis was the individual patient. Only one baseline clinical and laboratory assessment per patient was included in the dataset. No repeated measurements or multiple samples from the same patient were used for model development or validation, thereby preserving the assumption of independence.

### Data preprocessing and dataset splitting

Data preprocessing was performed to ensure consistency and minimize bias in model development. Missing data comprised less than 5% of the dataset and were handled using mean imputation for continuous variables and mode imputation for categorical variables. Continuous variables included in the predictive models were standardized as appropriate to reduce the influence of differences in numerical scale across predictors.

The full dataset was randomly divided into training, validation, and test sets at proportions of 70%, 15%, and 15%, respectively. To address class imbalance between relapse and non-relapse cases, the Synthetic Minority Oversampling Technique (SMOTE) was applied only to the training set after data splitting and before model fitting. The validation and test sets were kept untouched to avoid information leakage and to preserve the integrity of model evaluation.

### Multicollinearity and interaction assessment

Before logistic regression model fitting, multicollinearity among candidate predictors was formally assessed using variance inflation factors (VIFs). A VIF value <5 was considered indicative of acceptable collinearity. Particular attention was given to clinically related variables, such as prior relapse history and steroid resistance, that might share overlapping information. All evaluated predictors demonstrated VIF values within the acceptable range, indicating no problematic multicollinearity. Detailed VIF values are provided in **Supplementary Table 1**.

Potential interaction effects between clinically relevant predictors were also explored during logistic regression model development. Candidate interaction terms were selected on the basis of clinical plausibility and prior literature, with particular attention to combinations involving disease severity, treatment response, and relapse history. Each candidate interaction term was assessed by adding it individually to the main-effects model and evaluating changes in model fit and discrimination. Interaction terms were retained only if they materially improved model performance without compromising model stability or interpretability. No interaction term provided sufficient added value for inclusion in the final model, and therefore the final logistic regression model included main effects only.

### Model development

Three predictive models were developed to assess the risk of relapse within 12 months: a logistic regression model, a random forest classifier, and a deep learning-based neural network.

Logistic regression was used as the conventional statistical reference model. After collinearity assessment and interaction screening, all candidate baseline predictors were entered into a multivariable logistic regression model. Backward stepwise variable selection based on the Akaike information criterion (AIC) was then used to derive a parsimonious final model while preserving clinical interpretability and model stability.

The random forest model was developed as a non-linear ensemble learning approach capable of capturing complex interactions among predictors through multiple decision trees. The deep learning model was implemented as a multilayer perceptron (MLP) neural network designed to learn non-linear relationships within the baseline clinical dataset. To ensure comparability, all three models were developed using the same predefined baseline predictor set.

Model tuning was performed during model development to optimize predictive performance while reducing the risk of overfitting. Hyperparameters for the machine learning models were selected using the training and validation sets according to predefined tuning procedures. The test set was reserved exclusively for final model evaluation and was not used during model training or tuning.

### Model evaluation and validation

Model performance was assessed primarily by discrimination and classification accuracy. Discrimination was evaluated using the area under the receiver operating characteristic curve (AUC). Classification performance was evaluated using accuracy, sensitivity, specificity, F1-score, and positive predictive value (PPV) at a predefined probability threshold. Accuracy was defined as the proportion of correctly classified cases, sensitivity as the ability to correctly identify relapse events, and specificity as the ability to correctly identify non-relapse patients. The F1-score was used to provide a balanced summary of precision and sensitivity, particularly in the presence of class imbalance.

The final evaluation of all three models was performed on the independent test set. Performance in the training and validation sets was also recorded to facilitate comparison of model fitting, tuning, and generalizability. Calibration of the logistic regression model was additionally assessed using the Hosmer–Lemeshow goodness-of-fit test, as this method is applicable to parametric regression-based models.

### Feature importance and interpretability

Feature importance analysis was conducted as a *post hoc* interpretability procedure after model training. This analysis was used only to characterize the relative contribution of predictors to relapse prediction and did not influence variable selection, model fitting, or model evaluation. The purpose of this analysis was to improve clinical interpretability and to identify predictors that contributed most strongly to model performance.

### Sample size considerations

Sample size considerations differed across modeling approaches. For logistic regression, the overall cohort size of 562 patients provided an adequate number of relapse events to support multivariable modeling and stable parameter estimation. However, more complex machine learning approaches, including random forest and deep learning models, generally require larger datasets to achieve optimal generalizability. Accordingly, while the present dataset was considered sufficient for comparative model development and internal evaluation, external validation in larger independent cohorts will be required to confirm robustness and transportability.

## Results

### Baseline characteristics of the study population

**Figure 1:**
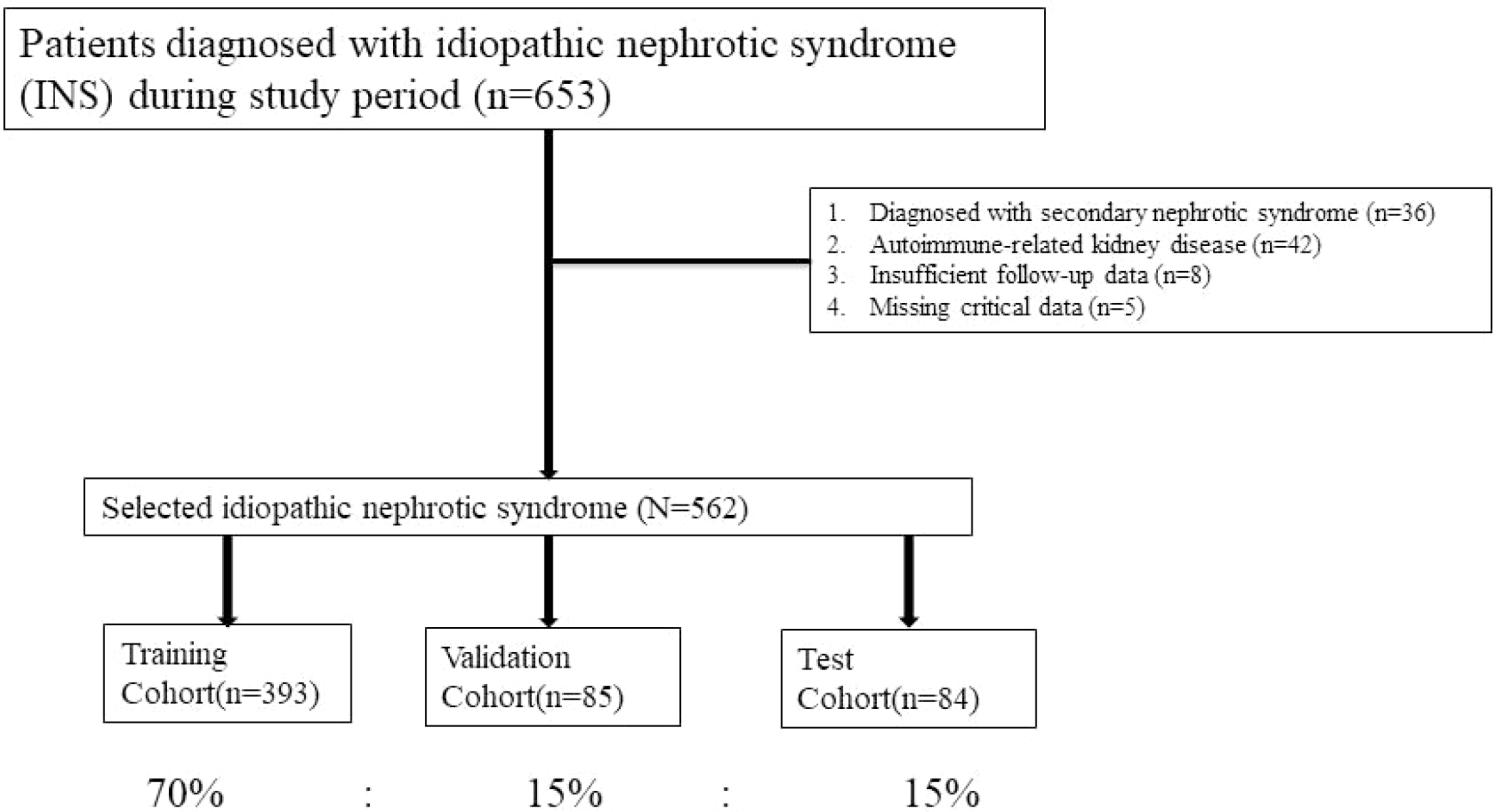
Flow chart for inclusion and exclusion of patients with idiopathic nephrotic syndrome.

A total of 562 adult patients with idiopathic nephrotic syndrome (INS) were included in this study. The dataset was randomly divided into training, validation, and test cohorts in a 70:15:15 ratio, resulting in 393 patients in the training set, 85 in the validation set, and 84 in the test set. The patient selection process is summarized in **Figure 1**.

In the training cohort, 64.4% of patients were aged ≥30 years, 29.0% had an ESR >20 mm/hour, 24.2% had a CRP >5 mg/dL, and 37.7% had nephrotic-range proteinuria (>3.5 g/day) at baseline. Steroid resistance was present in 33.1% of patients, and 27.7% had a history of prior relapse. Regarding baseline treatment, 34.1% received steroids alone, 39.2% received immunosuppressants, and 26.7% received combination therapy. During follow-up, relapse occurred in 18.0% of patients in the training cohort. No statistically significant differences in baseline characteristics were observed across the training, validation, and test cohorts (all p>0.05). Detailed baseline characteristics are presented in **Table 1**.

**Table 1:** Baseline characteristics of patients with idiopathic nephrotic syndrome (INS) in the training, validation, and test cohorts (n=562).

Variable		Training cohort (n=393)	Validation cohort (n=85)	Test cohort(n=84)	P-Value‡
Age (years)		32.8 ± 9.6	33.1 ± 9.4	32.9 ± 9.8	0.910
ESR> 20 mm/hour (%)					0.891
	No	279 (71.0)	61 (71.8)	60 (71.4)	
	Yes	114 (29.0)	24 (28.2)	24 (28.6)	
CRP > 5 mg/dL (%)					0.761
	No	298 (75.8)	65 (76.5)	63 (75.0)	
	Yes	95 (24.2)	20 (23.5)	21 (25.0)	
ACE > 52 U/L (%)					0.680
	No	285 (72.5)	61 (71.8)	59 (70.2)	
	Yes	108 (27.5)	24 (28.2)	25 (29.8)	
Proteinuria > 3.5 g/day (%)					0.544
	No	245 (62.3)	53 (62.4)	52 (61.9)	
	Yes	148 (37.7)	32 (37.6)	32 (38.1)	
Chronic diseases (%)					0.693
	No	269 (68.4)	58 (68.2)	57 (67.9)	
	Yes	124 (31.6)	27 (31.8)	27 (32.1)	
History of relapses (%)					0.723
	No	284 (72.3)	60 (70.6)	59 (70.2)	
	Yes	109 (27.7)	25 (29.4)	25 (29.8)	
Steroid resistance (%)					0.615
	No	263 (66.9)	58 (68.2)	57 (67.9)	
	Yes	130 (33.1)	27 (31.8)	27 (32.1)	
Use of immunosuppressants (%)					0.977
	No	134 (34.1)	30 (35.3)	29 (34.5)	
	Yes	259 (65.9)	55 (64.7)	55 (65.5)	
Smoking (%)					0.530
	No	278 (70.7)	58 (68.2)	57 (67.9)	
	Yes	115 (29.3)	27 (31.8)	27 (32.1)	
Hypertension (%)					0.993
	No	274 (69.7)	59 (69.4)	59 (70.2)	
	Yes	119 (30.3)	26 (30.6)	25 (29.8)	
Diabetes mellitus (%)					0.582
	No	305 (77.6)	65 (76.5)	63 (75.0)	
	Yes	88 (22.4)	20 (23.5)	21 (25.0)	
Dyslipidemia (%)					0.461
	No	278 (70.7)	58 (68.2)	57 (67.9)	
	Yes	115 (29.3)	27 (31.8)	27 (32.1)	
Body Mass Index (BMI > 25) (%)					0.419
	No	245 (62.3)	53 (62.4)	52 (61.9)	
	Yes	148 (37.7)	32 (37.6)	32 (38.1)	
eGFR < 60 ml/min (%)					0.501
	No	305 (77.6)	65 (76.5)	63 (75.0)	
	Yes	88 (22.4)	20 (23.5)	21 (25.0)	
First administered treatment option (%)					0.401
	Steroids	134 (34.1)	30 (35.3)	29 (34.5)	
	Immunosuppressants	154 (39.2)	33 (38.8)	32 (38.1)	
	Combination Therapy	105 (26.7)	22 (25.9)	23 (27.4)	
Recurrence (%)					0.951
	No	322 (82.0)	69 (81.2)	68 (81.0)	
	Yes	71 (18.0)	16 (18.8)	16 (19.0)	
First episode at baseline (%)					0.904
	No	109 (27.7)	25 (29.4)	25 (29.8)	
	Yes	284 (72.3)	60 (70.6)	59 (70.2)	
Relapse phenotype (%)					0.997
	Non-FRNS/SDNS	248 (63.1)	54 (63.5)	53 (63.1)	
	FRNS	92 (23.4)	19 (22.4)	19 (22.6)	
	SDNS	53 (13.5)	12 (14.1)	12 (14.3)	
Genetic screening performed (%)					0.999
	No	51 (13.0)	11 (12.9)	11 (13.1)	
	Yes	342 (87.0)	74 (87.1)	73 (86.9)	

Values in parentheses are percentages unless otherwise indicated.

P-values are derived from the appropriate statistical tests.

ESR, Erythrocyte Sedimentation Rate; CRP, C-Reactive Protein; ACE, Angiotensin-Converting Enzyme; Egfr, Estimated Glomerular Filtration Rate; BMI, Body Mass Index.

### Regression analysis of relapse risk factors

Univariate logistic regression analysis identified several baseline variables associated with relapse risk, including ESR > 20 mm/hour, CRP > 5 mg/dL, proteinuria > 3.5 g/day, chronic diseases, history of relapses, steroid resistance, use of immunosuppressants, smoking, dyslipidemia, BMI > 25, eGFR < 60 mL/min, and combination therapy.

In the multivariate logistic regression model, ESR > 20 mm/hour (OR = 1.49, 95% CI: 1.09-2.38, p = 0.022), CRP > 5 mg/dL (OR = 1.39, 95% CI: 1.02-1.93, p = 0.043), proteinuria > 3.5 g/day (OR = 1.85, 95% CI: 1.38-2.55, p = 0.001), history of relapses (OR = 2.27, 95% CI: 1.65-3.22, p < 0.001), steroid resistance (OR = 2.58, 95% CI: 1.78-3.92, p < 0.001), BMI > 25 (OR = 1.46, 95% CI: 1.05-2.12, p = 0.039), eGFR < 60 mL/min (OR = 1.65, 95% CI: 1.15-2.26, p = 0.003), and combination therapy (OR = 1.83, 95% CI: 1.25-2.33, p = 0.004) remained independently associated with an increased risk of relapse. In addition, baseline use of immunosuppressants showed a borderline-to-moderate association with relapse risk (OR = 1.48, 95% CI: 1.05-2.07, p = 0.011). Detailed results are presented in **Table 2**.

**Table 2:** Univariate and multivariate logistic regression analysis of factors associated with relapse in idiopathic nephrotic syndrome (INS).

Variable	Univariate analysis OR (95% CI)	P-value	Multivariate analysis OR (95% CI)	P-value	β (SE)
Age (per 1 year)	1.03 (0.98-1.11)	0.316	1.02 (0.97-1.12)	0.289	0.14 (0.18)
ESR > 20 mm/hour	1.89 (1.32-2.71)	0.001	1.49 (1.09-2.38)	0.022	0.44 (0.15)
CRP > 5 mg/dL	1.74 (1.22-2.48)	0.002	1.39 (1.02-1.93)	0.043	0.34 (0.16)
ACE > 52 U/L	1.32 (0.92-1.89)	0.117	1.27 (0.92-1.69)	0.235	0.21 (0.17)
Proteinuria > 3.5 g/day	2.12 (1.54-2.92)	<0.001	1.85 (1.38-2.55)	0.001	0.64 (0.19)
Chronic diseases	1.56 (1.09-2.24)	0.018	1.42 (0.92-1.99)	0.073	0.32 (0.17)
History of relapses	2.75 (1.99-3.81)	<0.001	2.27 (1.65-3.22)	<0.001	0.84 (0.17)
Steroid resistance	3.12 (2.26-4.32)	<0.001	2.58 (1.78-3.92)	<0.001	1.00 (0.18)
Use of immunosuppressants	1.88 (1.34-2.64)	0.001	1.48 (1.05-2.07)	0.011	0.43 (0.16)
Smoking	1.68 (1.17-2.41)	0.005	1.35 (0.95-1.88)	0.053	0.33 (0.17)
Hypertension	1.29 (0.92-1.82)	0.141	1.25 (0.92-1.83)	0.266	0.20 (0.16)
Diabetes mellitus	1.43 (0.99-2.06)	0.056	1.28 (0.92-1.86)	0.095	0.27 (0.17)
Dyslipidemia	1.52 (1.08-2.14)	0.015	1.38 (0.79-1.92)	0.156	0.24 (0.18)
BMI > 25	1.77 (1.25-2.51)	0.002	1.46 (1.05-2.12)	0.039	0.35 (0.17)
eGFR < 60 mL/min	1.98 (1.34-2.93)	<0.001	1.65 (1.15-2.26)	0.003	0.51 (0.18)
First administered treatment option					
Steroids (reference)	1.00	–	1.00	–	
Immunosuppressants	1.52 (1.09-2.12)	0.014	1.28 (0.91-1.99)	0.092	0.29 (0.17)
Combination therapy	1.98 (1.34-2.93)	<0.001	1.83 (1.25-2.33)	0.004	0.54 (0.19)

ESR, erythrocyte sedimentation rate; CRP, C-reactive protein; ACE, angiotensin-converting enzyme; eGFR, estimated glomerular filtration rate; BMI, body mass index.

### Predictive performance of machine learning models

**Figure 2:**
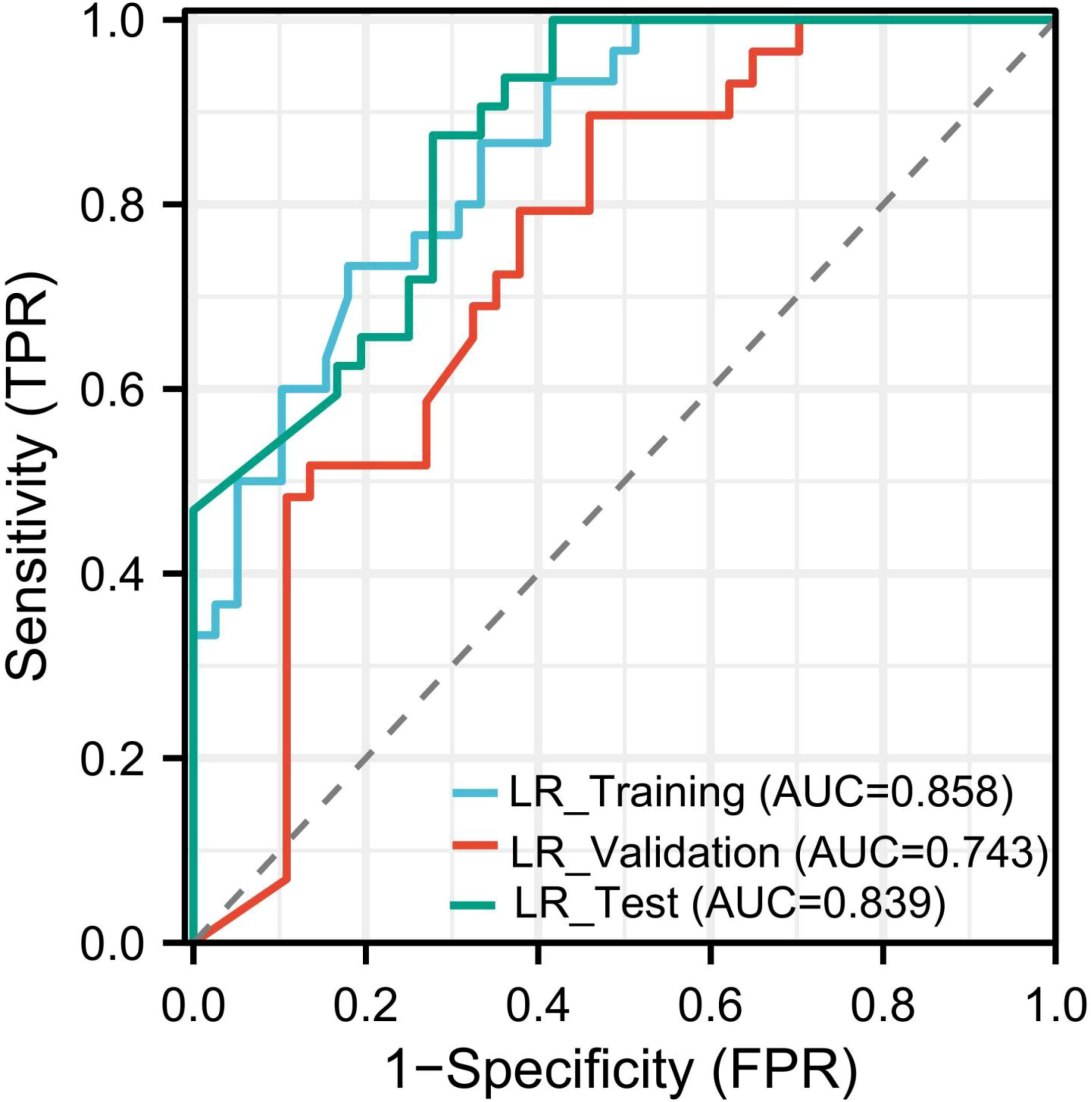
Performance of the logistic regression model for ROC curve.

A logistic regression model was constructed using baseline predictors selected through multivariable analysis. The model achieved AUC values of 0.858, 0.743, and 0.839 in the training, validation, and test cohorts, respectively (**Figure 2**).

**Figure 3:**
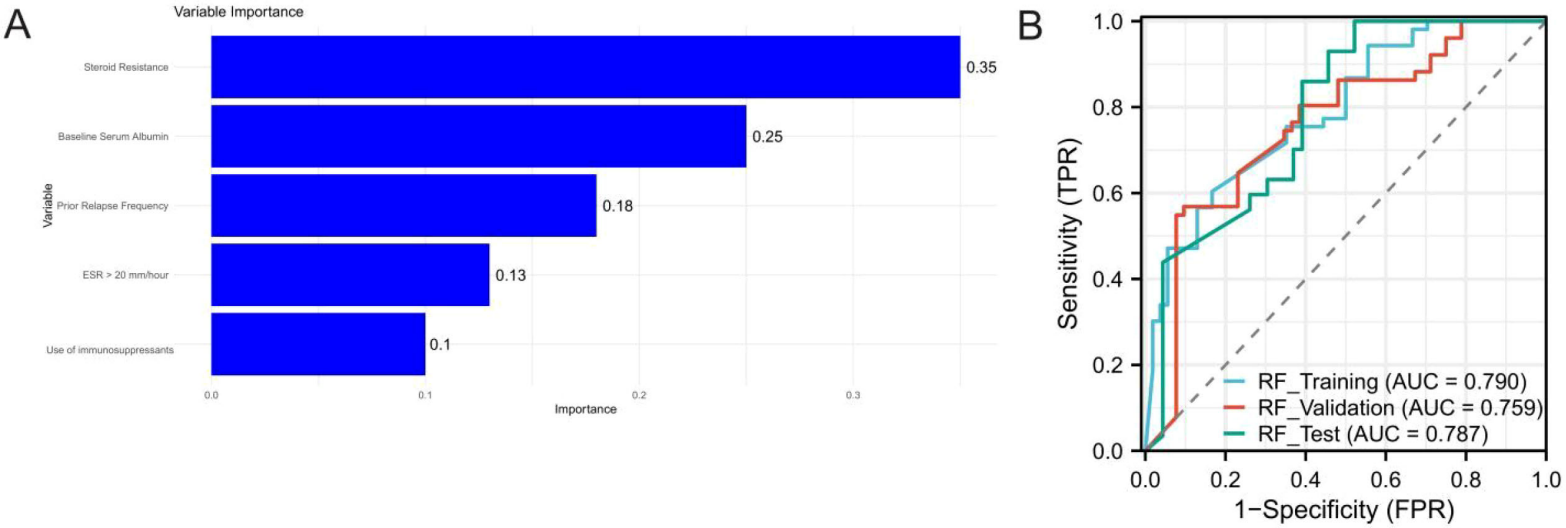
ROC curves of the Random Forest model on the training, validation and test sets. **(A)** represents the five most important variables, and **(B)** represents the ROC curve of the model on the training, validation and test sets.

For the random forest model, hyperparameter tuning identified an optimal configuration with 500 decision trees, a maximum depth of 12, and a minimum of 3 samples required for splitting. The model achieved AUC values of 0.790, 0.759, and 0.787 in the training, validation, and test cohorts, respectively (**Figure 3**).

**Figure 4:**
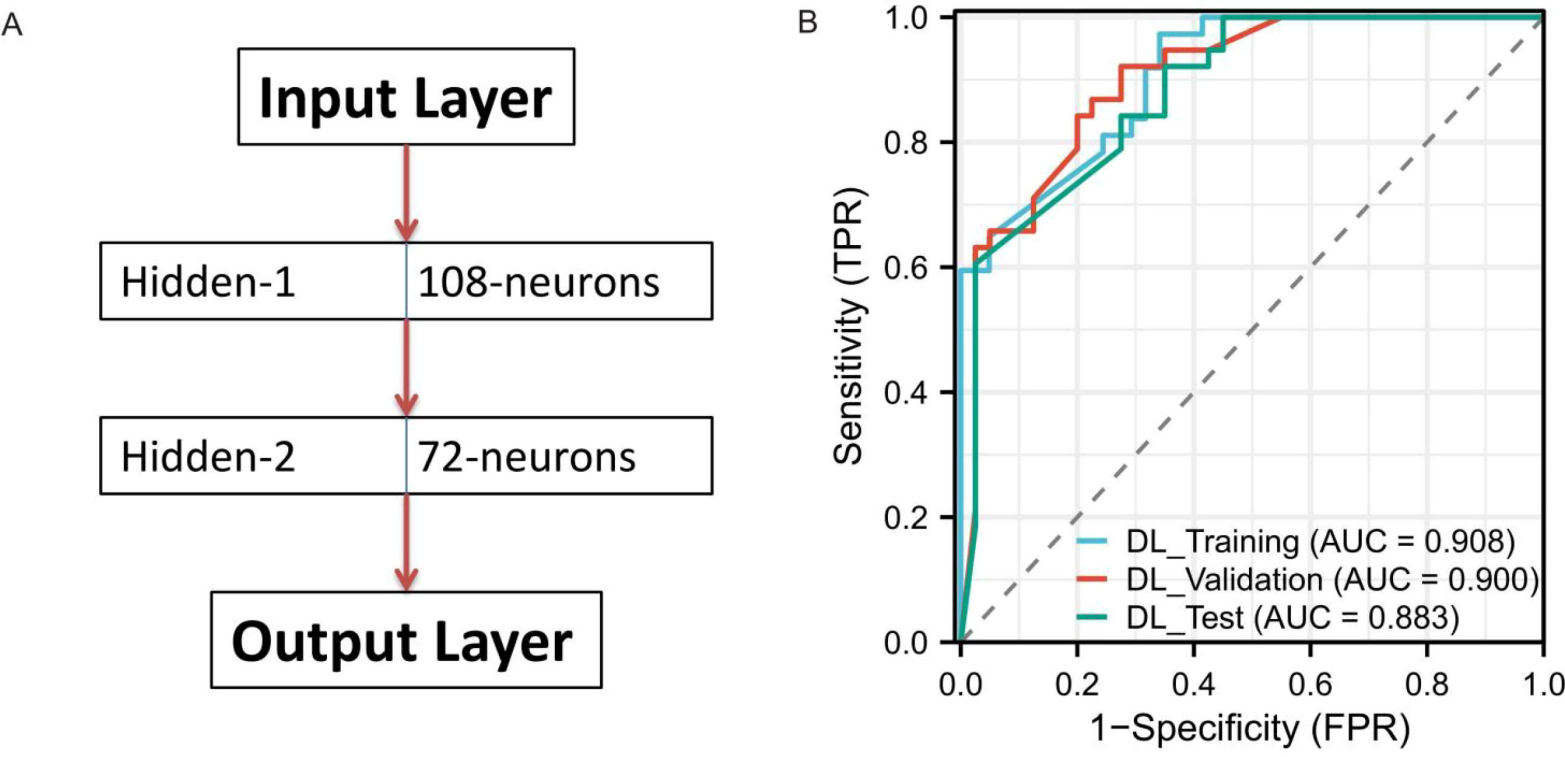
Performance of the Deep Learning Model. **(A)** represents the model architecture diagram, and **(B)** represents the ROC curve of the model on the training, validation and test sets.

The deep learning model was implemented as a multilayer perceptron with two hidden layers containing 108 and 72 neurons, respectively (**Figure 4A**). This model demonstrated the best overall performance, with AUC values of 0.908, 0.900, and 0.883 in the training, validation, and test cohorts, respectively (**Figure 4B**).

### Comparison of model performance

Among the three models, the deep learning model showed the best overall predictive performance. Its F1-scores were 0.915, 0.893, and 0.903 in the training, validation, and test cohorts, respectively, with corresponding AUC values of 0.908, 0.900, and 0.883.The logistic regression model showed intermediate performance, with F1-scores of 0.799, 0.772, and 0.793 and corresponding AUC values of 0.858, 0.743, and 0.839. The random forest model showed slightly lower but still acceptable performance, with F1-scores of 0.784, 0.762, and 0.785 and AUC values of 0.790, 0.759, and 0.787 across the three datasets. Detailed performance metrics are presented in **Table 3**.

**Table 3:** Evaluation indicators for each model.

Model	F1 Score	ROC-AUC	95% CI	PPV	Hosmer–Lemeshow
Logistic Regression					0.62
Training	0.799	0.858	0.760-0.892	0.78	
Validation	0.772	0.743	0.688-0.797	0.74	
Test	0.793	0.839	0.785-0.926	0.77	
Random Forest					
Training	0.784	0.790	0.712-0.880	0.76	
Validation	0.762	0.759	0.662-0.832	0.72	
Test	0.785	0.787	0.726-0.859	0.75	
Deep Learning					
Training	0.915	0.908	0.839-0.978	0.88	
Validation	0.893	0.900	0.834-0.919	0.85	
Test	0.903	0.883	0.805-0.948	0.87	

ROC, receiver operating characteristic; AUC, area under the curve; CI, confidence interval; PPV, positive predictive value.

These findings indicate that the deep learning model provided the strongest discrimination for predicting relapse in adult patients with idiopathic nephrotic syndrome, although all three models showed reasonable predictive utility. Results of the exploratory analyses including post-baseline immunosuppressive therapy are provided in the **Supplementary Table 2**.

## Discussion

Idiopathic nephrotic syndrome (INS) is a common glomerular disease characterized by severe proteinuria, hypoalbuminemia, hyperlipidemia, and edema. Although many patients achieve remission after initial treatment, relapse remains a major clinical problem and is associated with repeated treatment exposure, increased healthcare utilization, and a higher risk of chronic kidney disease progression and treatment-related complications (1, 2, 11, 12). In this context, the ability to identify patients at high risk of relapse using routinely available baseline clinical information is of clear clinical relevance. In recent years, machine learning (ML) techniques have emerged as promising tools for improving predictive modeling in medicine, yet their application to relapse prediction in INS remains relatively limited.

In the present study, we developed and compared three predictive models—logistic regression, random forest, and deep learning—to assess relapse risk in adult patients with INS. Overall, our findings showed that the deep learning model achieved the best predictive performance among the three approaches, whereas the logistic regression model showed intermediate performance and random forest showed relatively lower discrimination. These findings suggest that ML-based approaches, particularly deep learning, may improve relapse prediction when multiple clinical variables are considered simultaneously. At the same time, the predictive advantage observed in our dataset should be interpreted in a balanced manner. Logistic regression remained a clinically meaningful and interpretable baseline model, and the differences between models, although favoring deep learning, should not be overstated beyond the scope of the present data.

Our findings are broadly consistent with previous studies indicating that conventional regression-based approaches may be limited when complex relationships among predictors are present. Logistic regression remains valuable for identifying independent associations and for maintaining interpretability, but its predictive capacity may be constrained when clinical variables interact in nonlinear or multidimensional ways. This perspective is in line with previous reports in nephrology and related fields. Bierzynska et al. (13, 14) emphasized the challenges of relapse prediction in nephrotic syndrome and the limitations of traditional approaches in the setting of heterogeneous clinical patterns. Similarly, Cai et al. (15)reported that conventional regression models may be less effective for complex disease prediction when multiple interacting features are involved. Our results support these observations, as logistic regression was informative for risk factor assessment but did not achieve the same overall discriminative performance as the more flexible deep learning-based models.

Among the three evaluated models, the deep learning model demonstrated the best overall performance in our dataset. A plausible explanation for this finding is that multilayer neural networks are designed to capture nonlinear relationships and hidden feature representations that may not be adequately modeled by conventional regression approaches. In clinical conditions such as INS, where relapse is likely influenced by a combination of disease severity, treatment responsiveness, prior relapse burden, inflammatory activity, and baseline clinical heterogeneity, such modeling flexibility may provide an advantage. This interpretation is supported by previous work in nephrology and related areas. Lee et al. (16)reported that deep learning models outperformed traditional statistical methods in predicting kidney transplant rejection, illustrating the potential of neural network-based approaches in complex clinical prediction settings. Likewise, Barisoni et al. (17)demonstrated that deep learning substantially improved diagnostic performance in kidney biopsy image analysis compared with conventional classification techniques. Although those studies addressed different nephrology applications, they collectively support the broader notion that deep learning may be particularly useful when medical prediction tasks involve intricate and multidimensional data patterns. Our findings extend this idea to relapse prediction in adult INS.

Interpretability, however, remains an important challenge for the clinical adoption of complex ML models. One of the principal strengths of logistic regression is that it provides coefficients and odds ratios that are relatively straightforward to interpret clinically. By contrast, deep learning models are often regarded as “black-box” systems, which may limit physician confidence and reduce immediate acceptability in routine practice. For this reason, model performance alone should not be the sole criterion for model selection. Rather, predictive discrimination, interpretability, feasibility of implementation, and clinical context should all be considered together. In the present study, we therefore incorporated *post hoc* feature importance analysis to improve interpretability and to identify clinically relevant predictors contributing to relapse prediction.

The feature importance analysis highlighted several influential predictors, including steroid resistance, nephrotic-range proteinuria at baseline, prior relapse history/frequency, inflammatory activity, and immunosuppressant exposure. These findings are clinically plausible and are generally consistent with previous literature identifying disease activity and treatment responsiveness as major determinants of relapse risk in nephrotic syndrome (18). Among these factors, steroid resistance emerged as one of the strongest contributors to relapse prediction, which is concordant with previous studies showing that steroid-resistant patients are more likely to experience recurrent disease, treatment refractoriness, and poorer long-term renal outcomes (19). Steroid resistance may also reflect underlying biological heterogeneity, including more severe disease phenotypes and potential genetic susceptibility, both of which may complicate treatment response and increase relapse risk (20). In clinical practice, early identification of patients with steroid resistance may therefore be particularly important, as these individuals are more likely to require intensified monitoring and alternative therapeutic strategies (21). Another important issue in relapse prediction concerns the handling of treatment-related variables. In this study, baseline immunosuppressive therapy was included as a candidate predictor because it reflects treatment exposure and disease status at the time of baseline assessment. In contrast, post-baseline immunosuppressive therapy was not included in the primary predictive models, because treatment changes after baseline are inherently influenced by early clinical course and physician decision-making. Such variables may therefore reflect evolving disease response rather than baseline relapse susceptibility. For this reason, our models were designed specifically for baseline relapse risk prediction rather than treatment-effect estimation. Future studies incorporating longitudinal data structures, time-dependent covariates, or causal inference frameworks may be better suited to clarify the relationship between treatment modification and subsequent relapse.

It is also important to place our findings in the context of the broader literature evaluating ML in nephrology. Previous studies assessing relapse prediction in nephrotic syndrome and related chronic conditions have sometimes reported only modest differences between logistic regression and more complex ML approaches (22–25). Our results are not necessarily inconsistent with this literature. This suggests that while ML may offer additional discriminatory value, the magnitude of improvement can vary depending on the patient population, predictor set, outcome definition, and sample size. Accordingly, logistic regression remains a relevant benchmark model, particularly when interpretability and ease of clinical implementation are prioritized.

Several limitations of this study should be acknowledged. First, this was a retrospective single-center study, which may limit generalizability to other patient populations and clinical settings. Second, although the cohort size was sufficient for internal model development and comparison, more complex ML models generally benefit from larger datasets, and the current findings require external validation in independent cohorts. Third, some continuous variables were categorized using clinically established thresholds to improve interpretability and facilitate potential clinical application, but this approach may have reduced the amount of information available to the models. Fourth, although feature importance analysis was performed to improve interpretability, the deep learning model remains less transparent than logistic regression, which may hinder direct clinical adoption. Finally, because the study focused on baseline prediction of 12-month relapse, our results should not be interpreted as evidence regarding causal treatment effects or long-term outcome modification.

Despite these limitations, the present study provides a systematic comparison of logistic regression, random forest, and deep learning models for predicting relapse in adult INS using routinely available baseline clinical data. The findings suggest that deep learning may provide improved predictive discrimination relative to traditional and ensemble-based approaches in this setting, while logistic regression remains a useful and interpretable reference model. Taken together, these results support the potential value of ML-based approaches for relapse risk stratification in INS, while also underscoring the need for cautious interpretation, external validation, and further work on explainability before routine clinical implementation.

## Conclusion

In conclusion, among the three evaluated approaches, the deep learning model showed the best overall predictive performance for 12-month relapse in adult patients with idiopathic nephrotic syndrome, whereas logistic regression remained a clinically interpretable reference model with acceptable discrimination. These findings suggest that machine learning-based approaches, particularly deep learning, may provide useful support for relapse risk stratification in INS by capturing complex relationships among baseline clinical variables that are less easily modeled by conventional regression methods. At the same time, the advantages of more complex models should be interpreted with appropriate caution, and external validation in larger, independent, and more diverse cohorts will be necessary before routine clinical implementation. Future studies should also focus on improving model interpretability, validating performance prospectively, and developing practical tools that can facilitate responsible translation of these methods into nephrology care.

## Data Availability

The original contributions presented in the study are included in the article/**Supplementary Material**. Further inquiries can be directed to the corresponding author.
