# Supplementary material for: Development and evaluation of machine learning models for predicting relapse in idiopathic nephrotic syndrome
Source: Front Endocrinol (Lausanne). 2026 Apr 13;17:1687315. doi: 10.3389/fendo.2026.1687315 (PMC13111052; doi:10.3389/fendo.2026.1687315)
Supplement: Supplementary file 1 [file Table1.docx]

Supplementary Table 1. Variance inflation factors (VIFs) for candidate predictors included in the logistic regression model.

| **Predictor** | **Type** | **VIF** |
| --- | --- | --- |
| Age | Continuous | 0.88 |
| ESR > 20 mm/hour | Categorical | 1.21 |
| CRP > 5 mg/dL | Categorical | 1.34 |
| ACE > 52 U/L | Categorical | 0.96 |
| Proteinuria > 3.5 g/day | Categorical | 2.18 |
| eGFR < 60 ml/min | Categorical | 1.67 |
| BMI > 25 | Categorical | 1.12 |
| Smoking | Categorical | 0.79 |
| Hypertension | Categorical | 1.48 |
| Diabetes mellitus | Categorical | 1.26 |
| Dyslipidemia | Categorical | 1.58 |
| Chronic diseases | Categorical | 1.73 |
| Steroid resistance | Categorical | 2.41 |
| History of relapses (prior relapse) | Categorical | 2.63 |
| Use of immunosuppressants | Categorical | 1.94 |
| First administered treatment option (3-level) | Categorical | 2.09 |
