# Supplementary material for: Development and evaluation of machine learning models for predicting relapse in idiopathic nephrotic syndrome
Source: Front Endocrinol (Lausanne). 2026 Apr 13;17:1687315. doi: 10.3389/fendo.2026.1687315 (PMC13111052; doi:10.3389/fendo.2026.1687315)
Supplement: Supplementary file 2 [file Table2.docx]

Supplementary Table 2. Exploratory analysis including baseline and post-baseline immunosuppressive therapy across logistic regression, random forest, and deep learning models

| **Model** | **Baseline immunosuppressive therapy included** | **Post-baseline immunosuppressive therapy included** | **Test-set AUC**  **(95% CI)** | **Test-set F1-score** |
| --- | --- | --- | --- | --- |
| Logistic regression | Yes | No | 0.839 (0.785-0.926) | 0.793 |
| Logistic regression | Yes | Yes (time-fixed exploratory analysis) | 0.844 (0.784-0.912) | 0.798 |
| Random forest | Yes | No | 0.787 (0.726-0.859) | 0.785 |
| Random forest | Yes | Yes (time-fixed exploratory analysis) | 0.792 (0.721-0.856) | 0.784 |
| Deep learning | Yes | No | 0.883 (0.834-0.919) | 0.893 |
| Deep learning | Yes | Yes (time-fixed exploratory analysis) | 0.916 (0.805-0.956) | 0.904 |

**Abbreviations:** AUC, area under the receiver operating characteristic curve; CI, confidence interval.

**Note:** Post-baseline immunosuppressive therapy was included only in an exploratory time-fixed analysis and should not be interpreted as a causal treatment effect estimate. Because treatment modifications after baseline may reflect early disease course and clinical response, these analyses were performed for exploratory comparison only and were not part of the primary baseline prediction models. All other predictors, model structures, dataset partitions, and evaluation procedures were unchanged from the primary analysis.
